# Identification and In Silico Characterization of Novel *Helicobacter pylori* Glucose-6-Phosphate Dehydrogenase Inhibitors

**DOI:** 10.3390/molecules26164955

**Published:** 2021-08-16

**Authors:** Beatriz Hernández-Ochoa, Gabriel Navarrete-Vázquez, Rodrigo Aguayo-Ortiz, Paulina Ortiz-Ramírez, Laura Morales-Luna, Víctor Martínez-Rosas, Abigail González-Valdez, Fernando Gómez-Chávez, Sergio Enríquez-Flores, Carlos Wong-Baeza, Isabel Baeza-Ramírez, Verónica Pérez de la Cruz, Saúl Gómez-Manzo

**Affiliations:** 1 Programa de Posgrado en Biomedicina y Biotecnología Molecular, Escuela Nacional de Ciencias Biológicas, Instituto Politécnico Nacional, Ciudad de México 11340, Mexico; beatrizhb_16@comunidad.unam.mx (B.H.-O.); ing_vicmr@hotmail.com (V.M.-R.); 2 Laboratorio de Inmunoquímica, Hospital Infantil de México Federico Gómez, Secretaría de Salud, Ciudad de México 06720, Mexico; 3 Facultad de Farmacia, Universidad Autónoma del Estado de Morelos, Av. Universidad 1001, Chamilpa, Cuernavaca, Morelos 62209, Mexico; gabriel_navarrete@uaem.mx; 4 Departamento de Farmacia, Facultad de Química, Universidad Nacional Autónoma de México, Mexico City 04510, Mexico; rodaguayo@comunidad.unam.mx; 5 Laboratorio de Bioquímica Genética, Instituto Nacional de Pediatría, Secretaría de Salud, Ciudad de México 04530, Mexico; paulo.r1396@gmail.com (P.O.-R.); lauraeloisamorales@ciencias.unam.mx (L.M.-L.); 6 Posgrado en Ciencias Biológicas, Universidad Nacional Autónoma de México, Ciudad de México 04510, Mexico; 7 Departamento de Biología Molecular y Biotecnología, Instituto de Investigaciones Biomédicas, Universidad Nacional Autónoma de México, Ciudad de México 04510, Mexico; abigaila@iibiomedicas.unam.mx; 8 Laboratorio de Inmunología Experimental, Instituto Nacional de Pediatría, Ciudad de México 04530, Mexico; fergocha@gmail.com; 9 Cátedras CONACyT-Instituto Nacional de Pediatría, Secretaría de Salud, Ciudad de México 04530, Mexico; 10 Departamento de Formación Básica Disciplinaria, Escuela Nacional de Medicina y Homeopatía del Instituto Politécnico Nacional, Ciudad de México 07320, Mexico; 11 Laboratorio de EIMyT, Grupo de Investigación en Biomoléculas y Salud Infantil, Instituto Nacional de Pediatría, Secretaría de Salud, Ciudad de México 04530, Mexico; sergioenriquez@ciencias.unam.mx; 12 Laboratorio de Biomembranas, Escuela Nacional de Ciencias Biológicas, Instituto Politécnico Nacional, Ciudad de México 11340, Mexico; charlywong@icloud.com (C.W.-B.); isabelbaeza@yahoo.com (I.B.-R.); 13 Neurochemistry and Behavior Laboratory, National Institute of Neurology and Neurosurgery “Manuel Velasco Suárez”, Ciudad de México 14269, Mexico; veped@yahoo.com.mx

**Keywords:** *Helicobacter pylori*, glucose-6-phosphate dehydrogenase, inhibitors, docking, homology modeling, drug candidates

## Abstract

*Helicobacter pylori* (*H. pylori*) is a pathogen that can remain in the stomach of an infected person for their entire life. As a result, this leads to the development of severe gastric diseases such as gastric cancer. In addition, current therapies have several problems including antibiotics resistance. Therefore, new practical options to eliminate this bacterium, and its induced affections, are required to avoid morbidity and mortality worldwide. One strategy in the search for new drugs is to detect compounds that inhibit a limiting step in a central metabolic pathway of the pathogen of interest. In this work, we tested 55 compounds to gain insights into their possible use as new inhibitory drugs of *H. pylori* glucose-6-phosphate dehydrogenase (HpG6PD) activity. The compounds **YGC-1**; **MGD-1, MGD-2; TDA-1**; and **JMM-3** with their respective scaffold 1,3-thiazolidine-2,4-dione; *1H*-benzimidazole; 1,3-benzoxazole, morpholine, and biphenylcarbonitrile showed the best inhibitory activity (IC_50_ = 310, 465, 340, 204 and 304 μM, respectively). We then modeled the HpG6PD protein by homology modeling to conduct an in silico study of the chemical compounds and discovers its possible interactions with the HpG6PD enzyme. We found that compounds can be internalized at the NADP^+^ catalytic binding site. Hence, they probably exert a competitive inhibitory effect with NADP^+^ and a non-competitive or uncompetitive effect with G6P, that of the compounds binding far from the enzyme’s active site. Based on these findings, the tested compounds inhibiting HpG6PD represent promising novel drug candidates against *H. pylori*.

## 1. Introduction

*Helicobacter pylori* (*H. pylori*) is a Gram-negative bacterium that infects approximately 50% of the world’s population. It colonizes the human stomach, causing a broad spectrum of diseases ranging from gastritis and gastric ulcers to stomach cancer [1,2], the latter being the third leading cause of cancer death worldwide—with 90% of cases being related to *H. pylori* infection [3]. To survive in hostile environments such as that of stomach acid pH, *H. pylori* can secrete urease to generate ammonia and carbon dioxide, which locally neutralizes the stomach’s acidity [4]. Thus, the effective and efficient eradication of *H. pylori* infection could reduce its enormous negative consequences in humans. However, there are no exclusive drugs against *H. pylori*, and current treatments consist of combining two or three antibiotics (clarithromycin, amoxicillin, and metronidazole) with one proton pump inhibitor such as omeprazole [5,6]. However, the finding of *H. pylori* strains that are resistant to clarithromycin or metronidazole compromises these therapeutic strategies [7]. Therefore, proposals for new therapeutic targets that contribute to developing compounds with pharmacological potential against *H. pylori* are urgently needed.

**Figure 1:**
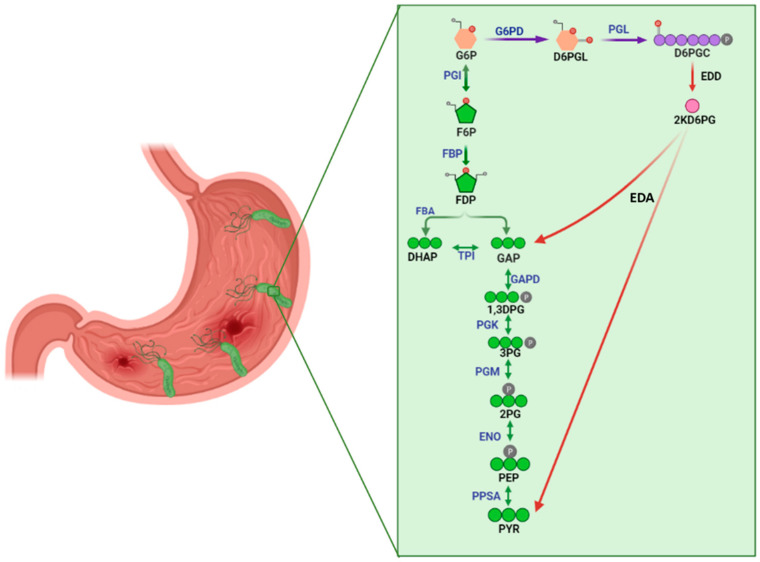
A graphical illustration of the central metabolism subsystem in the reconstructed network. The lilac arrows show the oxidative phase of the pentose phosphate pathway. The Entner–Doudoroff pathway is shown with red arrows, where glyceraldehyde-3-phosphate and pyruvate are obtained, and glycolysis is shown with green arrows. Metabolites are glucose 6 phosphate (G6P), fructose-6-phosphate (F6P), dihydroxyacetone phosphate (DHAP), glyceraldehyde-3-phosphate (GAP), 1,3-diphosphoglycerate (1,3DPG), 3-phosphoglycerate (3PG), 2-phosphoglycerate (2PG), phosphoenolpyruvate (PEP), pyruvate (PYR), 6-phosphogluconolactone (D6PGL), 6-phosphogluconate (D6PGC), 2-keto-3-deoxy-6-phosphogluconate (2KD6PG). Enzymes are phosphoglucose isomerase (PGI); fructose-1,6-bisphosphatase (FBP); fructose-1,6-bisphosphate aldolase (FBA); triosephosphate isomerase (TPI); glyceraldehyde-3-phosphate dehydrogenase (GAPD); phosphoglycerate kinase (PGK); phosphoglycerate mutase (PGM); enolase (ENO); phosphoenolpyruvate synthase (PPSA); glucose-6-phosphate dehydrogenase (G6PD); 6-phosphogluconolactonase (6PGL); 6-phosphogluconate dehydratase (EDD); and 2-keto-3-deoxy-6-phosphogluconate aldolase (EDA).

A complete genome study on the sequence of *H. pylori* strain 26695 confirms that it has a reduced genome size (1.7 Mb), and that this gastric pathogen has fewer complex metabolic pathways than other bacteria [8]. This bacterium has some of the genes that encode enzymes belonging to glycolysis or the Embden–Meyerhoff pathway (EMP); however, the genes that code for phosphofructokinase (PFK) and pyruvate kinase (PK) have not been identified. As the EMP is incomplete in *H. pylori*, it uses an alternate metabolic pathway to oxidize glucose, as the Entner–Doudoroff (ED) pathway is the major pathway of glucose catabolism under aerobic conditions. Additionally, it was reported that the genes belonging to the pentose phosphate pathway (PPP) are present, except for the third-gen of the PPP, the 6-phosphogluconate dehydrogenase (g6pdh); which in consequence excludes a complete oxidative phase [9]. The ED pathway (Figure 1) represents an offshoot of the oxidative branch of the PPP. The glucose-6-phosphate (G6P) was converted to 6-phosphogluconate (D6PGC) by the subsequent reaction of the two enzymes: glucose 6 phosphate dehydrogenase (G6PD) and 6-phosphogluconolactonase (6PGL) producing one NADPH/H^+^, the D6PGC is then converted to 2-keto-3-deoxy-6-phosphogluconate (2KD6PG) through the removal of a water molecule by the enzyme 6-phosphogluconate dehydratase (EDD) in the ED pathway. The 2KD6PG is then split by the enzyme 2KD6PG aldolase (EDA) into pyruvate and GAP [9] (Figure 1). GAP can then be catalyzed to pyruvate through the EMP pathway, producing two ATP and one NADH/H^+^.

To date, the PPP pathway in *H. pylori* has been poorly studied. However, some studies have reported the presence of metabolites of this metabolic pathway, strongly suggesting that its presence in *H. pylori* implies a possible mechanism to provide NADPH and ribose 5-phosphate for the synthesis of nucleic acids [10,11].

Thus, the PPP appears to be important in the metabolism of *H. pylori*, including the G6PD from *H. pylori* (HpG6PD). In addition to providing NADPH to the cell, it also allows obtaining 6-phosphogluconate, which can later enter the ED pathway and obtain glyceraldehyde and pyruvate, necessary in the metabolism of *H. pylori*. A key enzyme in PPP is G6PD; however, there are no reports of the HpG6PD enzyme. Therefore, in the present study, a first biochemical study of the HpG6PD enzyme is performed, and the analysis of compounds to inhibit this enzyme as a strategy for searching for new drugs against *H. pylori*. Furthermore, taking advantage of the structural and functional similarity with other G6PD proteins from bacteria, comparative models can be obtained that allow the possibility of carrying out and developing more extensive studies of inhibitors of this enzyme. A three-dimensional (3D) homodimer model of the G6PD protein from *H. pylori* was obtained in this work. Subsequently, the molecular docking and molecular dynamics simulation (MDS) were carried out with four chemical compounds that have the property of inhibiting the catalytic activity of the recombinant enzyme HpG6PD in different percentages, but this does not inhibit the human G6PD enzyme. Therefore, the HpG6PD inhibition could provide an approach to preventing and treating disease caused by *H. pylori*.

## 2. Results and Discussion

### 2.1. Construction of pET3aHisTEVP-zwf Vector and Purification of the Recombinant Protein HpG6PD

**Figure 2:**
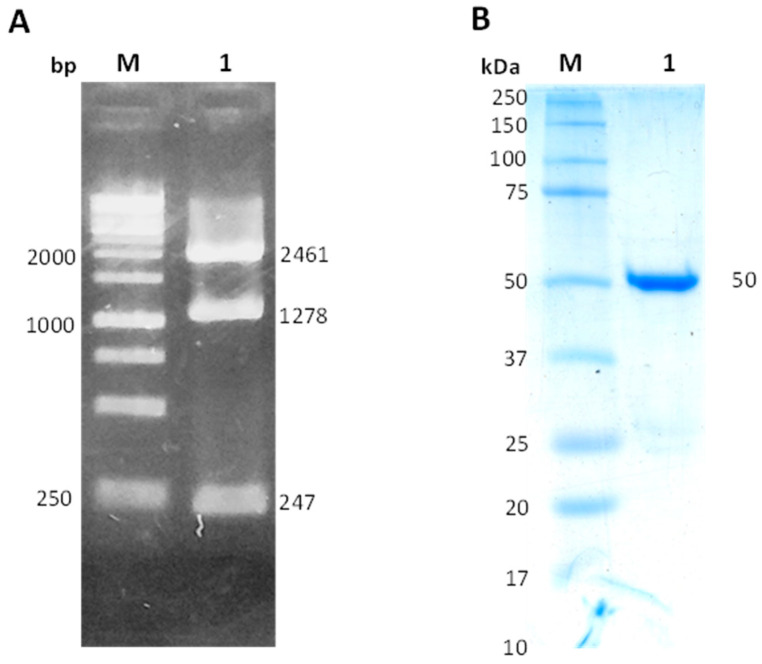
Construction of the vector pET3aHisTEVP-zwf and purification of the recombinant HpG6PD protein: (**A**) Analysis of the digestion with NdeI and BamHI enzymes in 1% agarose gel electrophoresis. M: GeneRuler 1 Kb Plus DNA molecular weight marker (Thermo Scientific^®^, Hudson, NH, USA). Line 1 Plasmid Puc57 digested with NdeI and BamHI enzymes; (**B**) Purification of the recombinant HpG6PD protein. M: molecular protein weight (MW) marker precision plus protein kaleidoscope standards from Bio-Rad (Hercules, CA, USA). Line 1: 20 micrograms of purified HpG6PD protein. The gels are representative of three independent experiments.

The commercial vector Puc57 with the zwf gene of *H. pylori* was digested with the restriction enzymes NdeI and BamHI. Figure 2A shows the digestion pattern, and a fragment of 1278 bp corresponding to the zwf gene of *H. pylori* was observed. In addition, two other bands of 2461 and 247 bp were observed. The first fragment corresponds to the Puc57 plasmid, and the second fragment corresponds to the internal *Nde*I restriction site to the *Nde*I cloning site of the *zwf* gene. The fragment of 1278 bp corresponding to the *zwf* gene was ligated to the pET3aHisTEVP vector, and the pET3aHisTEVP-zwf was obtained, which was later used to transform the competent *E. coli* BL21(DE3)Δzwf:kan^r^ cells for the expression of the recombinant HpG6PD protein. The HpG6PD protein was purified by Ni Sepharose high-performance affinity column (GE Healthcare). The 6xHis tag in the N-terminal was then removed using the site-specific protease TEVP as previously reported [12]. As seen in Figure 2B, the recombinant HpG6PD protein was detected as a single band with an apparent molecular weight (MW) of 50 kDa. This result is consistent with the theoretical MW of the monomeric form of the HpG6PD protein (49.45 kDa) according to the GenBank access number (ALM79592.1). In addition, this result is in agreement with those of previously reported for G6PDs proteins in other prokaryotes such as *Gluconacetobacter diazotrophicus*, *Leuconostoc mesenteroides*, *Thermotoga maritima*, and *Pseudomonas aeruginosa* [13,14,15,16], where G6PDs protein from 50 kDa was purified; which differed from Eukaryotic G6PDs proteins since they have MWs of approximately 60–75 kDa [17,18,19].

### 2.2. Selection of Inhibitors of HpG6PD of Helicobacter pylori

The previously purified HpG6PD enzyme was used to perform high-throughput screening assays to identify the chemical compounds that inhibited the enzyme activity. Previously, Preuss et al. [20] evaluated a library of approximately 50,000 chemical compounds and found five compounds that inhibited the homo sapiens G6PD (HsG6PD) activity. Based on the above, a library of 55 compounds with structural similarities to the previously reported molecules by Preuss and coworkers [20] was tested at a 400 μM concentration by a high-throughput screening assay. The results showed that 21 compounds reduced the activity of the HpG6PD by more than 40% (Supplementary Table S1). However, only six compounds showed inhibitory activity on the HpG6PD enzyme without significantly affecting the recombinant human HsG6PD enzyme activity. These compounds were grouped into four different families according to their structural similarities, named **YGC**, **JMM**, **TDA**, and **MGD**. The compounds’ structure and inhibition percentage at 400 μM are shown in Table 1.

The YGC compounds family inhibited HpG6PD activity from 44% to 84% of its initial activity and inhibited the HsG6PD from 0% to 18% of its initial activity. However, the compound with the higher specificity inhibitory activity on HpG6PD was YGC-1 with 84% inhibition. Regarding the **MGD** family, **MGD-1** and **MGD-2** inhibited HpG6PD activity at 50% and 55%, while the HsG6PD enzyme was only inhibited at 4% and 2%, respectively. The **TDA-1** compound inhibited the HpG6PD enzyme at 91%, while that the HsG6PD enzyme was only inhibited at 10% of its activity. The **JMM-3** compound inhibited the HpG6PD enzyme at 67% and in contrast, the HsG6PD by only 3%. Therefore, the degree of inhibition selectivity of **YGC1**, **YGC-3**, **MGD-1**, **MGD-2**, **TDA-1**, and **JMM-3** compounds could be used for the rational design of drugs as a new strategy to inhibit the G6PD enzyme of *H. pylori* without affecting the HsG6PD.

Finally, it is interesting to mention that notwithstanding the fact that **CNZ-3** and **CNZ-7** compounds inhibited HpG6PD, they also inhibited the HsG6PD enzyme at high levels (Supplementary Table S1), as previously reported by Ramírez-Nava et al. [21]. Therefore, these compounds were not used in in silico studies as they did not show selective inhibition.

**Figure 3:**
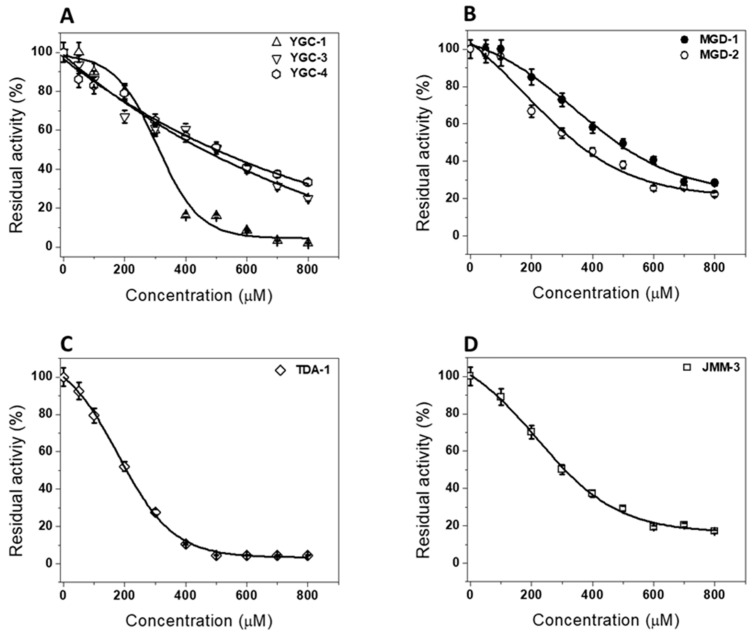
Inactivation assays of the HpG6PD enzyme with chemical compounds. The HpG6PD protein was adjusted at a final concentration of 200 μg/mL and incubated with increasing concentrations of the (**A**) **YGC** family; (**B**) **MGD** family; (**C**) **TDA-1** compound; and (**D**) **JMM-3** compound. The residual activity was determined after 2 h of incubation at 37 °C. The figure shows representative experiments performed in triplicate. The values represent the mean ± standard deviation from three independent experiments, and standard errors were lower than 5%.

Subsequently, we performed inactivation assays using different concentrations of compounds to analyze their concentration–activity effect. Figure 3 shows the residual activity of the HpG6PD enzyme after incubation for 2 h at 37 °C. As each of the compound’s concentrations was increased, the residual HpG6PD activity decreased in different degrees. For example, the exposure of the HpG6PD enzyme to **YGC-3** and **YGC-4** induced the abolition of the enzyme activity in a very similar manner (Figure 3A), the HpG6PD enzyme lost 80% of its activity at 800 µM, but when the enzyme was incubated with **YGC-1**, we observed a 91% loss in activity at 800 μM. On the other hand, **MGD-1** and **MGD-2** inactivated the HpG6PD enzyme, with 80% and 72% reductions, respectively, at the highest concentration tested (Figure 3B). In Figure 3C, the inactivation behavior is shown with the **TDA-1** compound, where a total loss of activity is reached by 500 µM. In contrast, **JMM-3** inhibited the HpG6PD enzyme at 80% with 800 µM (Figure 3D). From the plots of residual activity, we calculated the IC_50_ values; the efficacy of **YGC-1**, **MGD-2**, **TDA-1**, and **JMM-3** was particularly remarkable because the concentrations required to reduce the activity of the HpG6PD enzyme at 50% were 310, 340, 204, and 304 µM, respectively (Table 1). These compounds that showed that a high inhibition power in the HpG6PD enzyme possess the heterocycles 1,3-thiazolidine-2,4-dione and 1,3-benzoxazole scaffolds in their structure.

### 2.3. Homology Modeling of HpG6PD

**Figure 4:**
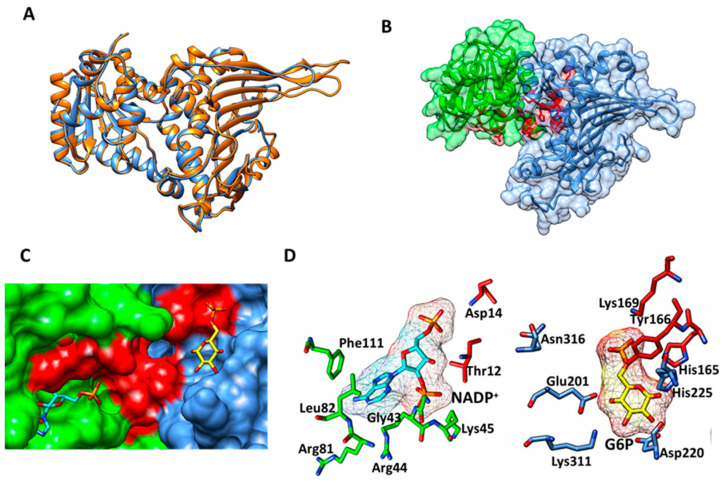
Structure generated of the G6PD protein from *Helicobacter pylori*: (**A**) structural alignment of HpG6PD (light blue color) with the G6PD of *L. mesenteriodes* (orange color); (**B**) surface representation of HpG6PD, the Rossmann domain is colored green and essential residues are shown as stick models—the coenzyme NADP^+^ and the substrate G6P binding sites are shown in red; (**C**) close-up of the probable binding site of both the NADP^+^ and G6P substrates; and (**D**) close-up of the substrate and coenzyme interactions with keys amino acids, NADP^+^ (cyan color), 6PG (yellow color).

To carry out the in silico study of the chemical compounds, it was necessary to obtain a model of the HpG6PD protein since it has not yet been crystallized. In this work, computational tools were applied that allowed us to obtain an adequate model of HpG6PD. The predicted and annotated sequence of G6PD from the *H. pylori* strain 29CaP (Identification code ALM79592.1) was used to perform a BLAST protein analysis against the Protein Data Bank (PDB), using a target database of 3D structures. The HpG6PD amino acids sequence showed the best score and the highest similarity (31%) with the G6PD of *Leuconostoc mesenteriodes* (PDB entry 1DPG) [14], 30% with the G6PD of *Mycobacterium avium* 104 (PDB entry 4LGV) [22], and 29% with the G6PD of *Trypanosoma cruzi* (PDB entry 6D23) [23]. To corroborate the results obtained with the BLAST protein, we used the SWISS-MODEL server [24] to identify possible molds to build the HpG6P model. This algorithm indicated that the PDB entry 1DPG was one of the suggested templates with 31% identity and with the best GMQE and QSQE scores with values of 0.72 and 0.55, respectively. Based on the results obtained with the BLASTP and SWISS-MODEL, we selected the 1DPG structure as the template to build the final model of HpG6PD using the Modeller 9.24 program [25]. To analyze the reliability of the representative model of HpG6PD, we aligned the predicted model with LmG6PD (PDB entry 1DPG), and an RMSD value of 0.725 Å was obtained for 405 Cα atoms, and the Q-score value was 0.828 (Figure 3A). In addition, the energy minimization of HpG6PD using the locPREFMD algorithm was performed (locPREFMD-FeigLab (msu.edu)) [26]. As expected, structural changes in some unstructured loops as well as some side-chains were observed after energy minimization; where a Ramachandran plot was achieved with 88.8%, 9.9%, 1.0%, and 0.3% of residues were in the most favored regions, additionally allowed regions, generously allowed regions and disallowed regions, respectively (Supplementary Figure S1). The N-terminal (amino acids 1–164) was found to contain the b-a-b Rossmann type folding domain, where the binding sites of NADP^+^ and b-d-glucose-6-phosphate (G6P) are located (Figure 4B). Moreover, it contains the conserved hexapeptide fragment 10-GATGDLA-16 (the number of amino acids corresponding to the HpG6PD protein of *H. pylori*) characteristics of the G6PD family of proteins that facilitate the binding to the catalytic NADP^+^ coenzyme. This fragment sequence was previously reported in human G6PD and is present in all the characterized G6PDs proteins [27]. Finally, the other two sequence fragments were identified. The sequence fragment 134-EKPLG-138, which contains Pro136, is involved in the correct positioning of the G6P substrate, and the sequence fragment 162-RIDHYLGKK-170, which contains the catalytic lysine residue (K169). The substrate-binding site was predicted from the PDB 1E77 structure of G6PD of *Leuconostoc mesenteroides*, co-crystallized with the NADP^+^ coenzyme and G6P substrate [28]. To predict the binding site of NADP^+^ and G6P, the HpG6PD model was in structural alignment with LmG6PD, and the amino acids involved in the binding site were identified (Figure 4C). For NADP^+^, the residues Thr12, Asp14, Gly43, Arg44, Lys45, Arg81, Leu82, and Phe111 were selected; while for G6P, the critical binding site residues His165, Tyr166, Lys169, Glu201, Asp220, His225, Lys311, and Asn316 were selected (Figure 4D).

### 2.4. Molecular Docking Studies with HpG6PD

**Figure 5:**
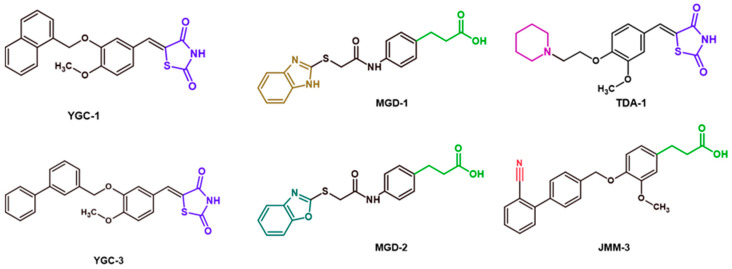
Chemical structures of the compounds used in the blind molecular docking: 1,3-thiazolidine-2,4-dione ring is shown in blue; 1*H*-benzimidazole ring is shown in coffee; 1,3-benzoxazole ring is shown in green; piperidine ring is shown in purple; and the butanoic acid group is shown in green. The chemical structures were generated with the ChemSketch program.

To predict the binding affinities of **YGC-1**, **YGC-3**, **MGD-1**, **MGD-2**, **TDA-1**, and **JMM-3** compounds that most reduced the activity of HpG6PD without significantly affecting the activity of the recombinant HsG6PD (Table 1) that we used, we performed a molecular blind docking analysis of these compounds with the HpG6PD model. The structure of the compounds is shown in Figure 5. The docking was performed considering the entire surface topology of the HpG6PD protein in the monomeric form. This analysis allowed us to predict the type of inhibition over the HpG6PD protein, inferring secondary/tertiary structure alterations and thermal stability analysis.

**Figure 6:**
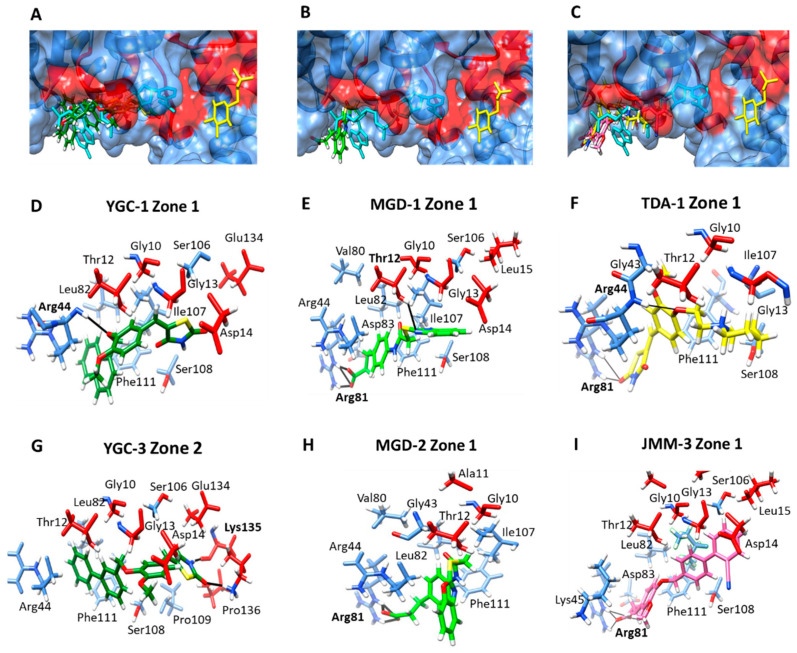
Molecular docking of the compounds in zone 1 on the HpG6PD model. In a general view of the binding cavities with the HpG6PD protein of (**A**) **YGC-1** and **YGC-2**, (**B**) **MGD-1** and **MGD-2**, and (**C**) **TDA-1** and **JMM-3**. The NADP^+^ and G6P molecules are shown in cyan and yellow color, respectively; (**D**,**G**) zoom on the interactions of **YGC-1** and **YGC-3**; (**E**,**H**) zoom on the interactions of **MGD-1** and **MGD-2**; (**F**,**I**) zoom on the interactions of **TDA-1** and **JMM-3** ligands with the HpG6PD model. The H-bond interactions are shown in black, and the amino acid residue is marked in bold.

The in silico study suggested a similar binding mode for all the compounds in a zone near the binding site of the NADP^+^ coenzyme (Figure 6A–C). In this pocket, six amino acids in common—Gly10, Thr12, Gly13, Asp14, Leu82, and Phe111 (the amino acid number correspond to the G6PD sequence from *H. pylori*; as shown in Table 2)—were identified to be interacting with the compounds. The **YGC-1** compound interacted with Arg44 by an H-bond and its methoxy radical, and ten nonpolar contacts were found (Figure 6D); the most stable protein–ligand complex showed a ΔG = −7.67 kcal/mol. In comparison, **YGC-3** formed two H-bonds between the Lys135 and a ketone group of the thiazolidine ring of this ligand. Twelve nonpolar contacts were found (ΔG = −8.18 kcal/mol) (Figure 6G). Regarding the **MGD-1**, it was internalized and formed three hydrogen bonds with Arg81 and its carboxylic acid radical, which also formed an H-bond with Thr12 and benzimidazole ring (Figure 6E), while **MGD-2** could only form three H-bonds with its carboxylic acid group and the amino acid Arg81 (Figure 6H). Furthermore, the **TDA-1** compound has in its structure a 1,3-thiazolidine-2,4-dione ring and showed an inhibition value of 61%, formed two hydrogen bonds with Arg81 and a hydrogen bridge with Arg44 (Figure 6F), and the most stable conformer showed a ΔG = −6.21 kcal/mol. Finally, the blind molecular docking of **JMM-3** on HpG6PD revealed the formation of three H-bonds with Arg81 and eleven nonpolar contacts (Figure 6I). **JMM-3**, the most stable protein–ligand complex, showed a ΔG = −8.55 kcal/moll. These results revealed that the **YGC-1**, **YGC-3**, **MGD-1**, **MGD-2**, **TDA-1**, and **JMM-3** probably affect the correct binding of catalytic NADP^+^ because they interact with four residues and the conserved heptapeptide 10-GATGDLA-17 involved in the correct positioning of the NADP^+^ [27]. Moreover, we could suggest that these compounds have a competitive inhibition for the NADP^+^ substrate. In contrast, for the G6P substrate, the inhibition could be non-competitive or uncompetitive because the binding site of these compounds is far from the G6P binding site (Figure 6A–C).

**Figure 7:**
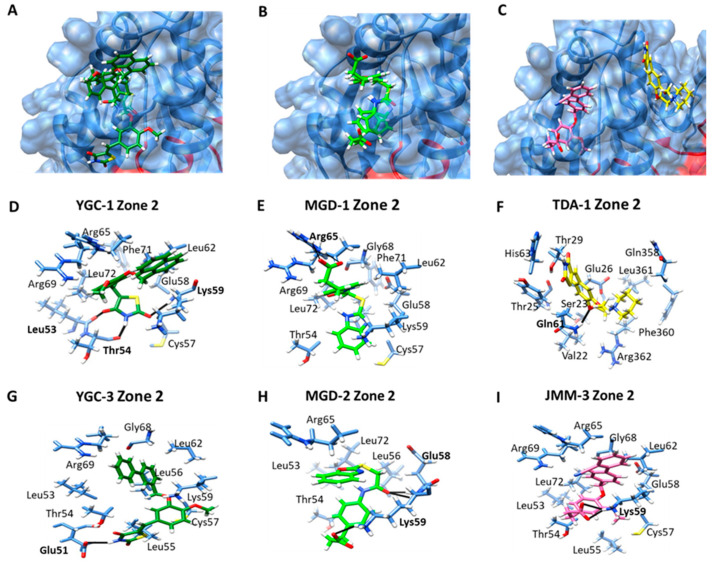
Molecular docking of compounds in zone 2 on the HpG6PD model. A general view of the binding cavities with the HpG6PD protein of (**A**) **YGC-1** and **YGC-2**; (**B**) **MGD-1** and **MGD-2**; and (**C**) **TDA-1** and **JMM-3**; (**D**,**G**) zoom on the interactions of **YGC-1** and **YGC-3**; (**E**,**H**) zoom on the interactions of **MGD-1** and **MGD-2**; and (**F**,**I**) zoom on the interactions of **TDA-1** and **JMM-3** ligands with the HpG6PD model. The H-bond interactions are shown in black, and the amino acid residue is marked in bold.

The docking also revealed a second zone of interaction for all compounds, which was found far from the active site (Figure 7A–C). **YGG-1** formed an H-bond with Lys59 and the 2-ketone of the benzimidazole ring. The 4-ketone formed a bond with Leu53, and a third H-bond was formed between the nitrogen from the thiazolidine ring and Thr54. Additionally, seven nonpolar contacts were determined, and 45% of the poses were obtained in the same pocket of HpG6PD (Figure 7D), where the most stable protein–ligand complex showed a ΔG = −8.05 kcal/mol; while **YGC-3** interacts with the HpG6PD protein by an H-bond with Glu51 and the nitrogen from thiazolidine ring, and nine nonpolar were found (Figure 7G). Regarding the docking assay with the **MGD** family, we found that **MGD-1** forms two hydrogen bonds between its carboxylic acid radical and Arg65 (Figure 7E). In contrast, the **MGD-2** compound forms an H-bond with the carboxylic acid radical and Lys59. Moreover, two H-bonds were observed between Glu58 and the oxygen of its acetamide (Figure 7H). Respecting **TDA-1**, a localized zone of interaction was found near previous compounds, where **TDA-1** formed an H-bond with Gln61 and the methoxy radical (Figure 7F). Finally, **JMM-3** also showed affinity for the same zone and can form two H-bonds with the carboxylic acid radical and Lys59 (Figure 7I).

**Figure 8:**
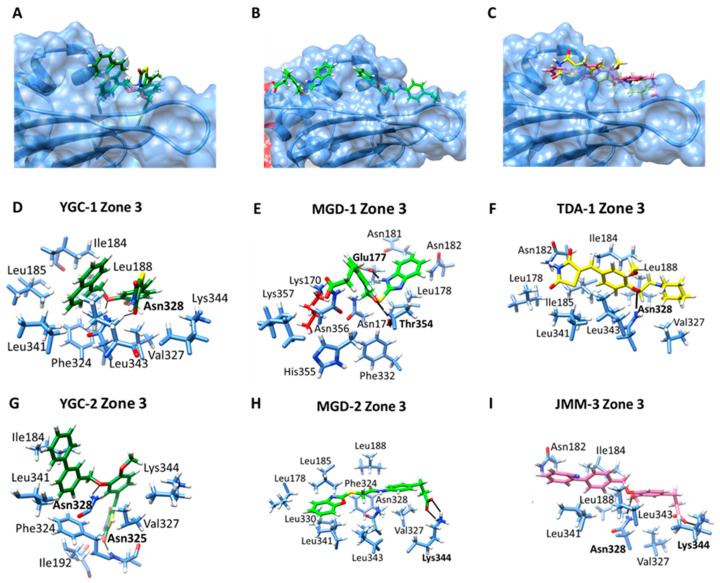
Molecular docking of compounds in zone 3 on the HpG6PD model. A general view of the binding cavities with the HpG6PD protein of (**A**) **YGC-1** and **YGC-2**; (**B**) **MGD-1** and **MGD-2**; and (**C**) **TDA-1** and **JMM-3**; (**D**,**G**) zoom on the interactions of **YGC-1** and **YGC-3**; (**E**,**H**) zoom on the interactions of **MGD-1** and **MGD-2**; and (**F**,**I**) zoom on the interactions of **TDA-1** and **JMM-3** ligands with the HpG6PD model. The H-bond interactions are shown in black, and the amino acid residue is marked in bold.

Finally, the blind molecular docking of all compounds on the HpG6PD revealed a third principal zone of interaction with the HpG6PD protein (Figure 8A–C). The **YGG-1** compound forms an H-bond between the naphthalenyl methoxy and the Asn328 amino acid. Additionally, an H-bond between the dione group and Ans328, and eight nonpolar contacts, and the 45% of the poses were obtained in the same pocket of HpG6PD (Figure 8D), where the most stable protein–ligand complex showed a ΔG = −7.16 kcal/mol; while **YGC-3** interacts with the HpG6PD protein by two H-bonds with Asn325 and Asn328 and six nonpolar contacts were found (Figure 8G). Regarding the **MGD** family, docking found that **MGD-1** forms a hydrogen bond with Glu177 and the benzimidazole ring. Additionally, a hydrogen bond with Thr354 and oxygen from the amide group was observed (Figure 8E). In contrast, the **MGD-2** compound forms two H-bonds between the Lys344 and the carboxylic group (Figure 8H). The **TDA-1** ligand showed affinity, one H-bond was formed with the Ans328, and eight nonpolar contacts were found (Figure 8F). Finally, the **JMM-3** ligand formed a hydrogen bridge with its carboxylic group and Lys344, as well as a hydrogen bridge between the methoxy group and Asn328 (Figure 8I).

According to the in silico study results, all the compounds evaluated by molecular docking also have the property to bind far from the active site of the enzyme (zone 2 and zone 3), so the type of enzyme inhibition could probably be uncompetitive or non-competitive. Moreover, it is interesting to note that non-competitive and uncompetitive inhibition was previously reported in HsG6PD, where alterations on the secondary/tertiary structure and thermal stability (global stability of the protein) were observed. Therefore, these compounds, which reduce the HpG6PD activity, could be considered a crucial characteristic for developing drugs that are increasingly safe, adequate, specific, and effective in treating diseases.

### 2.5. MD Simulation Characterization of HpG6PD-Ligand Complexes

**Figure 9:**
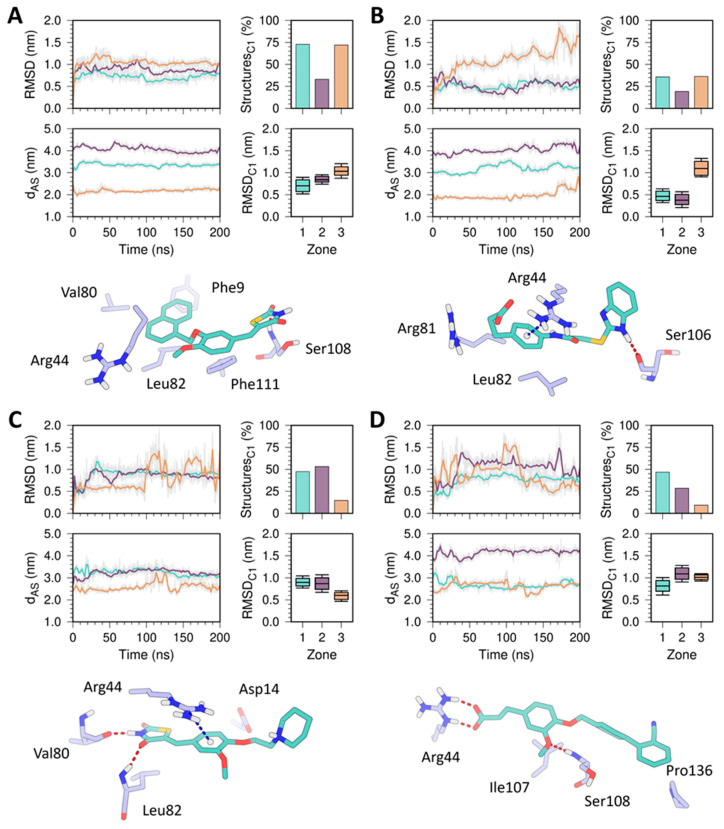
MD simulation analysis of compounds (**A**) **YGC-1**; (**B**) **MGD-1**; (**C**) **TDA-1**; and (**D**) **JMM-3** in Zones 1 (cyan), 2 (purple), and 3 (orange) on the HpG6PD model. The plots in the figure show the RMSD over time (200 ns) of each compound after the least-square fit to the HpG6PD backbone (top left), the distance between the geometry centers of the ligand and HpG6PD active site (dAS, bottom left), and the percentage of structures (top right) and RMSD distribution (bottom right) of the most populated clusters (C1). The tridimensional structures beneath the plots depict the compound interaction of the C1 cluster in zone 1 on the HpG6PD model. The H-bond and cation–π interactions are shown in red and blue dashed lines, respectively.

The experimental and molecular docking results demonstrated that the compounds **YGC-1**, **MGD-1**, **TDA-1**, and **JMM-3** exhibited higher inhibitory activities against HpG6PD and shared similar interaction sites on its structure. Nevertheless, an understanding of the dynamic behavior of the protein–ligand complexes is required to determine the interaction profile and stability of the compounds at the potential binding sites. Therefore, we carried out 200 ns all-atom MD simulations of the four molecules bound to the three different zones on HpG6PD (Figure 9). The RMSD of the ligands after a least-square fit to the HpG6PD backbone showed that all the compounds suffer substantial changes in their initial binding modes during the simulation (RMSD > 0.2 nm). These pose rearrangements that could be associated with the high solvent exposure of the binding sites and many flexible bonds in the compound structures. Despite the significant conformational changes and displacement observed through the simulations, none of the compounds were positioned less than 1.0 nm (dAS < 1.0 nm) from the center of geometry of the active site. To group the ligand conformations obtained from the simulations and determine each compound’s most probable binding modes, we performed an RMSD-based clustering analysis using a cutoff value of 0.25 nm and the last 150 ns of the simulation trajectories. Herein, we retrieved the cluster with the highest number of structures (Cluster 1, C1) from the simulations of the three different zones and computed the distribution of the RMSD values to have a quick comparison between groups. Overall, the analysis showed that compounds bound to Zone 1 were those with the most populated C1 cluster, the slightest fluctuation in the RMSD values, and the lowest difference to the initial position. Interestingly, the most representative protein–ligand complex of each C1 cluster confirmed the conservation of the ligands’ interaction with Arg44. These representative snapshots showed that compounds **YGC-1** and **JMM-3** formed van der Waals and H-bond interactions, respectively, with the Arg44 side chain (Figure 9A,D), while the phenyl group of compounds **MGD-1** and **TDA-1** displayed a cation–π interaction with the guanidine residue (Figure 9B,C). Other residues that played a vital role in the protein–ligand interaction were Leu82, Ser106, and Ser108, forming van der Waals interactions or H-bonds with the ligands. Despite the structural diversity among the molecules studied, we can assume that Zone 1 in HpG6PD could be the most likely binding site for these compounds to exert their inhibitory activity.

## 3. Materials and Methods

### 3.1. pET3aHisTEVP-zwf Vector Construction

The *zwf* gene encoding the G6PD protein was purchased from Genscript Company (Piscataway, NJ, USA). The *zwf* gene sequence of the *H. pylori* strain 29CaP was obtained from the GenBank with the accession number ALM79592.1. Restriction sites *Nde*I and *BamH*I were added at the 5′ and 3′ end, respectively; the plasmid pUC-*zwf* was digested with these enzymes to release the *zwf* gene. The product obtained from the digestion was analyzed on a 1% agarose gel electrophoresis. The gel was stained with an ADN Midori Green Advance (NIPPON Genetics Europe, Dueren, Germany) and visualized on a MultiDoc-Itequipment (UVP, Upland, San Bernardino, CA, USA). Then, the *zwf* gene fragment was purified using the GeneJET Gel Extraction Kit (Thermo Scientific, Hudson, NH, USA). Subsequently, it was digested with *Nde*I and *BamH*I restriction enzymes and ligated to the expression vector pET3aHisTEVP producing the plasmid named pET3aHisTEVP-*zwf*. Then, this plasmid was used to transform competent *E. coli* BL21(DE3)Δ*zwf*:kan^r^ cells to express the HpG6PD recombinant.

### 3.2. Expression and Purification of the Recombinant G6PD of Helicobacter Pylori

The *E. coli* BL21(DE3)Δ*zwf*:kan^r^ cells containing the expression vector pET3aHisTEVP-*zwf* were used to inoculate 2 L of Luria–Bertani (LB) medium supplemented with 100 μg/mL ampicillin (Sigma Aldrich, St. Louis, MO, USA) and 50 μg/mL kanamycin (Sigma Aldrich, St. Louis, MO, USA). The bacterial culture was grown by nine h at 37 °C and 180 rpm. Later, when the culture reached an optical density (OD) of 0.8 (600 nm) was induced with 0.5 mM isopropyl-β-d-1-thiogalactopyranoside (IPTG) (Thermo Fisher Scientific, Hudson, NH, USA) and incubated for 12 h at 25 °C and 180 rpm. Subsequently, the cells were concentrated by centrifugation at 6000 rpm at 4 °C for 10 min. Then, the cell button was suspended in lysis buffer (0.1 M Tris-HCl, pH 7.6, 3 mM MgCl_2_, 0.5 mM phenylmethylsulfonyl fluoride (PMSF) (Thermo Fisher Scientific, Hudson, NH, USA), 0.1% β-mercaptoethanol, and 5% glycerol), and disrupted by sonication [29]. After that, the debris were centrifuged at 10,000 rpm at 4 °C for 30 min, and the crude extract (supernatant) was used to purify HpG6PD.

The crude extract was applied to a Ni Sepharose^®^ high-performance column (GE Healthcare, Chicago, IL, USA), pre-equilibrated with binding buffer pH 8.0 (50 mM Tris, 150 mM NaCl plus 5% glycerol). The column was washed with 10 column volumes of wash buffer (50 mM Tris-HCl, pH 8.0, 150 mM NaCl, 50 mM imidazole, 2 mM DTT, and 5% glycerol). Subsequently, the HpG6PD protein was eluted with the binding buffer solution plus 250 mM imidazole [12]. The imidazole was removed from the HpG6PD protein by five consecutive dilutions with binding buffer and concentrated with a microcon-30 kDa centrifugal filter unit (Millipore, Burlington, MA, USA). According to previous reports, the 6xHis tag region present in the N-terminus of the HpG6PD protein was eliminated by incubating it with the tobacco etch virus protease (TEVP) [12]. Finally, the HpG6PD recombinant purity was analyzed using 10% SDS-PAGE gels [30], and stained with Colloidal Brilliant Coomassie Blue R-250. The protein concentration was determined using Lowry et al. [31], and using bovine serum albumin as standard. The HpG6PD protein was used immediately in a high-screen assay.

Enzymatic activity was spectrophotometrically determined following the reduction of NADP^+^ at 340 nm. The standard reaction mix contains 50 mM Tris-HCl, pH 7.6, 3 mM MgCl_2_, 200 µM glucose 6 phosphate and 100 µM NADP^+^. The reaction was started with the addition of 1 µg of the purified HpG6PD. One unit of activity was defined as the amount of HpG6PD that catalyzes the oxidation of 1 µmol of glucose 6 phosphate per minute.

### 3.3. Selection of Inhibitors of HpG6PD of Helicobacter pylori

A chemical library of 55 compounds was evaluated through high-throughput screening (HTS) assays to determine those compounds with the ability to reduce the catalytic activity of the recombinant HpG6PD. Dr. Gabriel Navarrete-Vázquez from the Pharmacy Faculty of the Autonomous University of the State of Morelos provided the chemical compounds [32]. These compounds were selected based on the previously reported chemical inhibitors of human G6PD [21], prepared at a final concentration of 400 μM in 5% dimethyl sulfoxide (DMSO) (Sigma Aldrich, St. Louis, MO, USA), incubated with 0.2 mg/mL of HpG6PD (200 ng total) at 37 °C for two hours, and the HpG6PD residual activity was measured. In addition, the HpG6PD protein was incubated with 5% DMSO and used as a control. The compounds that showed more than 40% inhibition was selected and analyzed by molecular docking and molecular dynamics simulation (MDS).

### 3.4. Homology Modeling and Comparison of HpG6PD

The sequence of the protein HpG6PD strain 29CaP was obtained from GenBank with accession number (ALM79592.1), and it was submitted to the BLAST tool from NCBI to identify the closely related homologs, using a database of the Protein Data Bank, the matrix BLOSUM 62, and the Blastp algorithm. In addition, the search for homologs was carried out on the SWISS-MODEL server to determine the template for constructing the G6PD monomeric model for *H. pylori*. The crystal structure of the G6PD of *Leuconostoc mesenteroides* (LmG6PD) (PDB entry 1DPG chain A) [14] was selected as the mold for homology modeling. HpG6PD was modeled based on the crystallographic information of LmG6PD using MODELLER 9.24 software [25]. Ten models were generated, and the best model was selected according to the global model quality estimation (GMQE), quaternary structure quality estimate (QSQE), and qualitative model energy analysis (QMEAN) statistical parameters. The predicted HpG6PD structure was validated by structure alignment with LmG6PD using Chimera 1.14.2 software [33] to predict the Q-score and the root mean square deviation (RMSD), which were calculated by aligning the mold Gs6PGD and model constructed HpG6PD. If the Q-score and RMSD values are high and low, respectively, then the structure is acceptable. In addition, energy minimization of HpG6PD using the locPREFMD algorithm was performed (locPREFMD-FeigLab (msu.edu)) [26]. The graphical representations were performed with the Chimera software. Additionally, the PDBsum tools [34] were used for constructing the Ramachandran plot to visualize the backbone dihedral angles ψ against ϕ of the amino acid residues in the model structure.

### 3.5. Blind Molecular Docking

We performed blind docking using the SwissDock Server (http://www.swissdock.ch/docking, accessed on 7 May 2021) to identify all possible interactions of compounds on the HpG6PD protein [35]. Here, we used the G6PD model from *H. pylori* to add the hydrogen to the HpG6PD structure, and the atomic coordinates of HpG6PD were submitted to the MolProbity server (http://molprobity.biochem.duke.edu/, accessed on 5 May 2021) [36], and system energy minimization was also performed. First, the 3D structures of the compounds were prepared in ACD/ChemSketch software. Then, the ligand structures were energy-minimized by the UCSF Chimera software 1.14.2, and a protonated state was considered if the compounds had an ionic group. Finally, the docking performed in the SwissDock server generates all possible binding modes for each ligand; and the most favorable binding modes at a given pocket are clustered. The predictions file provided Cluster Rank/Element Full Fitness and estimated the binding free energy ΔG. In each experiment, a total of 256 conformers was obtained per ligand. The docking results were loaded into and analyzed in the Chimera software 1.14.2. The affinity energies, the tridimensional configuration, the formation of hydrogen bonds, specific atoms involved, and the distance between them were analyzed to select the most stable binding for each compound.

### 3.6. Molecular Dynamics Simulations

To evaluate the stability and dynamic behavior of the docked ligands on HpG6PD, we carried out 200 ns molecular dynamics (MD) simulations employing the AMBER99SB-ILDN [37] force field implemented in GROMACS 2019.6 software [38]. The ligands’ topologies and coordinates were generated using the ACPYPE [39] package with the AM1-BCC [40] model. The protein–ligand complexes were solvated with water (TIP3P model) in a cubic box and neutralized with 0.15 M NaCl. All systems were energy minimized using the steepest descent algorithm and equilibrated for 1.0 ns under canonical (NVT) and isothermal-isobaric (NPT) ensembles. Holonomic constraints for all bonds comprising hydrogen atoms were applied using the LINCS algorithm [41]. The v-rescale thermostat [42] was used to set the temperature at 300 K, and pressure was fixed at 1.0 bar with the Parrinello–Rahman barostat [43]. Lennard–Jones potential and short-range electrostatic interactions’ cutoffs were set to 1.2 nm, while long-range electrostatic interactions were computed using the Particle Mesh Ewald (PME) approach.

GROMACS 2019.6 built-in tools were used to compute the root-mean-square deviation (RMSD) of the ligands after a least-square fit to the HpG6PD backbone and the clustering analysis based on the ligand position of the last 150 ns (RMSD < 0.25 nm). The distance between the geometry centers of the ligand and HpG6PD active site was calculated with in-house Python scripts using the MDAnalysis [44] libraries. Plots were constructed with Gnuplot v5.0 [45].

## 4. Conclusions

The search for new drugs to combat existing diseases produced by *H. pylori* in humans is a priority. One of the strategies used for this purpose is looking for enzyme inhibitors involved in pathogen metabolism. Therefore, we focused on finding and testing compounds to inhibit the G6PD of *H. pylori* as a strategy for the rational design of drugs against this bacterium. We found seven compounds that showed HpG6PD’s selective inhibition. Throughout the in silico assays, it was predicted that **YGC-1**, **YCG-3**, **MGD-1**, **MGD-3**, **TDA-1**, and **JMM-3** are molecules that can be internalized at the NADP^+^ binding site and form hydrogen bridges with different amino acids. Therefore, they probably exert a competitive inhibitory effect with NADP^+^ while exerting an uncompetitive or non-competitive inhibitory effect with G6P. In addition, they showed interactions far from the active site of the protein. For the final reasons, the studied compounds can be proposed as HpG6PD inhibitors, and the compounds found represent promising starting points of optimization against the HpG6PD protein and may be promising novel drug candidates.

## Figures and Tables

**Table 1:** Compounds showing an inhibition more significant than 40% in the HpG6PD enzyme at a final concentration of 400 µM.

Compounds	Chemical Structure	HpG6PD Inhibition (%) at (400 μM)	HsG6PD Inhibition (%) at (400 μM)	HpG6PD IC_50_
**YGC-1**	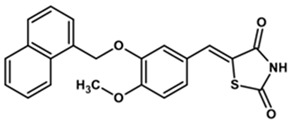	84	8	310
**YGC-3**	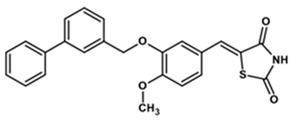	50	0	383
**YGC-4**	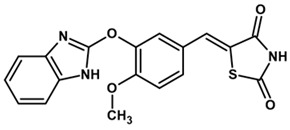	44	18	500
**MGD-1**	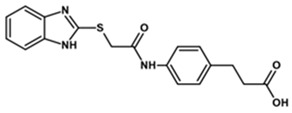	50	4	465
**MGD-2**	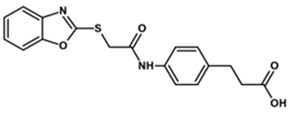	55	2	340
**TDA-1**	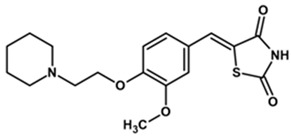	91	10	204
**JMM-3**	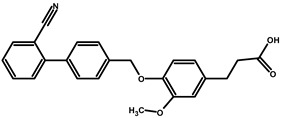	67	3	304

**Table 2:** Interactions of compounds with HpG6PD. The amino acids in bold are the hydrogen bond interactions.

Compound	Interactions	ΔG (kcal/mol)
**G6P**	His165, Tyr166, Lys169, Glu201, Asp220, His225, Lys311, Ans316	
**NADP^+^**	Thr12, Asp14, Gly43, Arg44, Lys45, Arg81, Leu82, Phe111	
**YGC-1**	*Zone 1*: Gly10, Thr12, Gly13, Asp14, **Arg44**, Leu82, Ser106, Ile107, Ser108, Phe111, Glu134.	−7.67
	*Zone 2*: **Leu53**, **Thr54**, Cys57, Glu58, Lys59, Leu62, Arg65, Arg69, Phe71, Leu72	−8.05
	*Zone 3*: Ile184, Leu185, Leu188, Phe324, Val327, **Asn328**, Leu341, Leu343, Lys344	−7.16
**YGC-3**	*Zone1*: Gly10, Thr12, Gly13, Asp14, Arg44, Leu82, Ser106, Ser108, Pro109, Phe111, Glu134, **Lys135**, Pro136.	−8.18
	*Zone 2*: **Glu51**, Leu53, Thr54, Leu55, Leu56, Cys57, Lys59, Leu62, Gly68, Arg69.	−7.87
	*Zone 3*: Ile184, Ile192, Phe324, Asn325, Val327, **Asn328**, Leu341, Lys344.	−7.81
**MGD-1**	*Zone 1*: Gly10, **Thr12**, Gly13, Asp14, Leu15, Arg44, Val80, **Arg81**, Leu82, Asp83, Ile107, Ser108, Phe111	−8.13
	*Zone 2*: Thr54, Cys57, Glu58, Lys59, Leu62, **Arg65**, Gly68, Arg69, Phe71, Leu72.	−8.20
	*Zone 3*: Lys170, Asn174, Glu177, Leu178, Asn181, Asn182, Phe332, **Thr354**, His355, Asn356, Lys357.	
**MGD-2**	*Zone 1*: Gly10, Ala11, Thr12, Gly43, Arg44, Val80, **Arg81**, Leu82, Ile107, Phe111.	−9.15
	*Zone 2*: Leu53, Thr54, Leu56, **Glu58**, **Lys59**, Arg65, Leu72.	−9.07
	*Zone 3*: Leu178, Leu185, Leu188, Phe324, Val327, Asn328, Leu330, Leu341, Leu343, **Lys344**	−8.16
**TDA-1**	*Zone 1*: Gly10, Thr12, Gly13, Gly43, **Arg44**, **Arg81**, Ile107, Ser108, Phe111	−6.21
	*Zone 2*: Val22, Ser23, Thr25, Glu26, Thr29, **Gln61**, His63, Gln358, Phe360, Leu361, Arg362.	−7.21
	*Zone 3*: Leu178, Asn182, Ile184, Ile185, Leu188, Val327, **Asn328**, Leu341, Leu343.	−6.25
**JMM-3**	*Zone* 1: Gly10, Thr12, Gly13, Asp14, Leu15, Lys45, **Arg81**, Leu82, Asp83, Ser106, Ser108, Phe111.	−8.55
	*Zone 2*: Leu53, Thr54, Leu55, Cys57, Glu58, **Lys59**, Leu62, Arg65, Gly68, Arg69, Leu72	−8.28
	*Zone 3*: Asn182, Ile184, Leu188, Val327, **Asn328**, Leu341, Leu343. **Lys344**.	−9.05

## Data Availability

Not applicable.

## Supplementary Materials

The following are available online, Supplementary Figure S1: Ramachandran plot of the homology modelled G6PD from *Helicobacter pylori*. Supplementary Table S1. Selection of inhibitors of HpG6PD of *Helicobacter pylori* at a final concentration of 400 μM.

## Sample Availability

Samples of the compounds are not available from the authors.
