# Supplementary material for: Identification and In Silico Characterization of Novel Helicobacter pylori Glucose-6-Phosphate Dehydrogenase Inhibitors
Source: Molecules. 2021 Aug 16;26(16):4955. doi: 10.3390/molecules26164955 (PMC8401736; doi:10.3390/molecules26164955)
Supplement: Supplementary file 1 [file molecules-26-04955-s001.zip › Supplementary Material/Supplementary Figure S1.pdf]

Supplementary Figure S1. Ramachandran plot of the homology modelled G6PD from *Helicobacter pylori*.

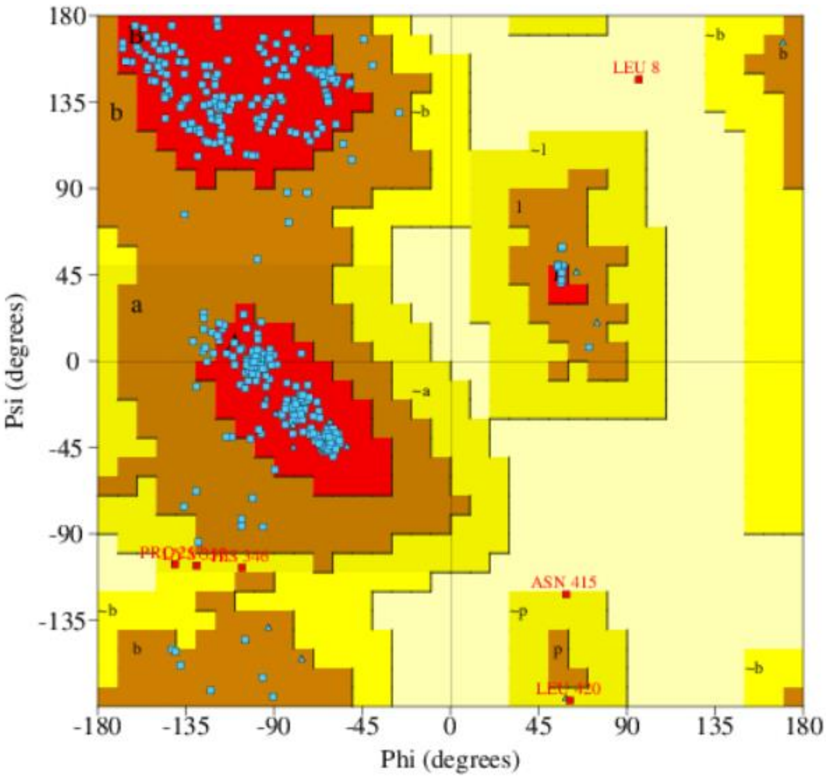

PROCHECK statistics

1. Ramachandran Plot statistics

```
                                              No. of
                                             residues    %-tage
                                              ------     ------
Most favoured regions      [A,B,L]             350       88.8%
Additional allowed regions [a,b,l,p]            39        9.9%
Generously allowed regions [~a,~b,~l,~p]         4        1.0%
Disallowed regions         [XX]                  1        0.3%*
                                               ----      ------
Non-glycine and non-proline residues           394      100.0%


Number of end-residues (excl. Gly and Pro)       2
Number of glycine residues (shown as triangles) 14
Number of proline residues                      15
                                               ----
Total number of residues                        425
```

Based on an analysis of 118 structures of resolution of at least 2.0 Angstroms and R-factor no greater than 20.0 a good quality model would be expected to have over 90% in the most favoured regions [A,B,L].
