# Supplementary material for: Identification and In Silico Characterization of Novel Helicobacter pylori Glucose-6-Phosphate Dehydrogenase Inhibitors
Source: Molecules. 2021 Aug 16;26(16):4955. doi: 10.3390/molecules26164955 (PMC8401736; doi:10.3390/molecules26164955)
Supplement: Supplementary file 1 [file molecules-26-04955-s001.zip › Supplementary Material/Supplementary Table S1.pdf]

Supplementary Table S1. Selection of inhibitors of HpG6PD of *Helicobacter pylori* at a final concentration of 400 μM.

Chemical compound	HpG6PD inhibition (%) at [400 μM]	HsG6PD inhibition (%) at [400 μM]
CCG-1	38	0
CCG-2	25	0
CCG-3	32	19
YGC-1	84	8
YGC-2	3	0
YGC-3	50	0
YGC-4	44	18
CCM-1	13	46
CCM-2	0	0
CCM-4	56	47
JMM-1	36	61
JMM-2	100	81
JMM-3	67	3
TDA-1	91	10
TDA-2	25	21
TDA-3	30	1
TDA-4	31	14
TDA-5	25	17
TDA-6	38	20
TDA-7	31	16
AGR-1	13	4
AGR-2	30	17
AGR-3	32	20
AGR-4	25	16
AGR-5	11	19
MGD-1	50	4
MGD-2	55	2
MGD-3	28	29
MGD-4	36	19
MGD-5	37	14
MGD-14	31	27
DMG-1	37	33
DMG-2	4	3
DMG-3	9	24
CNZ-15	23	20
CNZ-16	34	31
CNZ-17	35	0
CNZ-18	14	9
CNZ-21	27	0
CNZ-2	30	29
CNZ-3	88	92
CNZ-5	19	29
CNZ-6	35	30

CNZ-7	70	68
CNZ-8	58	43
CMC-1	59	34
CMC-2	30	9
CMC-3	36	20
MCC-7	42	11
TRZ-0	14	36
TRZ-1	31	30
TRZ-2	24	21
TRZ-3	19	0
TRZ-4	29	26
TRZ-5	0.9	7
